# Supplementary material for: Factors associated with delayed diagnosis of appendicitis in adults: A single-center, retrospective, observational study
Source: PLoS One. 2022 Oct 20;17(10):e0276454. doi: 10.1371/journal.pone.0276454 (PMC9584535; doi:10.1371/journal.pone.0276454)
Supplement: Attachment — Submitted filename: Response to Reviewers.docx [file pone.0276454.s002.docx]

Thank you very much for reviewing our manuscript and offering valuable advice.

We appreciate the invaluable comments that the reviewers and editors provided, which we are confident have helped us improve the manuscript. We have provided point-by-point responses to each of the comments and describe the related revisions below. We have indicated changes via red characters in the revised manuscript.

Thank you again for your thorough and supportive peer review.

We look forward to hearing from you.

On behalf of the co-authors,

Sincerely,

Taku Harada

Division of General Medicine, Showa University Koto Toyosu Hospital,

5-1-38 Toyosu Koto-ku, Tokyo, 135-8577, Japan

hrdtaku@gmail.com

■Additional Editor Comments:

The authors state that delayed diagnosis rates have only been reported as negative appendectomy rates. This is categorically not true - there are several previous studies that report delayed diagnosis rates as rates of missed diagnosis on an initial visit, generally < 5%. Please provide some comparative commentary on why your delay rates are so different. Is this a difference in health systems?

Response:

Thank you for pointing out this critical issue.

The frequency of diagnostic errors in appendicitis is vast, we think it is influenced by differences in healthcare systems and definitions of diagnostic errors as you mentioned.

Also, a pediatric study, similar to ours, that defined diagnostic delay based on the diagnosis at the first consult reported 66% diagnostic delay (JAMA Netw Open. 2021;4(8):e2122248).

In response to your comment, we have added a description in discussion part as follows:

*(P15 L287-93)*

*On the other hand, although a pediatric study, Michelson et al. in their multicenter case-control study of diagnostic errors in appendicitis, in which the definition of diagnostic delay was strictly defined as failure to diagnose at the first consult as in our study, reported that 63.0% of patients had a diagnostic delay [42]. Despite similar definitions of diagnostic delay, we believe that this large difference in the frequency of diagnostic errors in appendicitis is also influenced by situativity, including patient age and differences in health care systems [43].*
