# Supplementary material for: Factors associated with delayed diagnosis of appendicitis in adults: A single-center, retrospective, observational study
Source: PLoS One. 2022 Oct 20;17(10):e0276454. doi: 10.1371/journal.pone.0276454 (PMC9584535; doi:10.1371/journal.pone.0276454)
Supplement: Attachment — Submitted filename: Response to Reviewers.docx [file pone.0276454.s003.docx]

Thank you very much for reviewing our manuscript and offering valuable advice.

We appreciate the invaluable comments that the editors provided, which we are confident have helped us improve the manuscript. We have provided point-by-point responses to each of the comments and describe the related revisions below.

We also submitted it to a proofreader who corrected the wording to make it more appropriate

We have indicated changes via red characters in the revised manuscript.

Thank you again for your thorough and supportive peer review.
We look forward to hearing from you.
On behalf of the co-authors,


Sincerely,
Taku Harada
Division of General Medicine, Showa University Koto Toyosu Hospital,
5-1-38 Toyosu Koto-ku, Tokyo, 135-8577, Japan
[hrdtaku@gmail.com](mailto:hrdtaku@gmail.com)

■Journal Requirements:

Please review your reference list to ensure that it is complete and correct. If you have cited papers that have been retracted, please include the rationale for doing so in the manuscript text, or remove these references and replace them with relevant current references. Any changes to the reference list should be mentioned in the rebuttal letter that accompanies your revised manuscript. If you need to cite a retracted article, indicate the article’s retracted status in the References list and also include a citation and full reference for the retraction notice.


*Response:*

*Thank you for pointing out the important issue.*

*We apologize for the duplication of No. 14 and No. 27 in the references during the Revise process.

Reference No.27 has been deleted.
Reference No. 44 has been corrected.
Reference No.41 has been newly added.*

■Additional Editor Comments:

I am happy with the changes and how you have contextualized the findings. The Michelson study showed that 63% of patients who had a revisit had a delay in diagnosis. Most patients do not have revisits. So, 63% of the small fraction of patients with a revisit leading to an appendicitis diagnosis had a delay. Overall, delay rates are still < 5% among all children diagnosed with appendicitis. As an example, Mahajan et al in JAMA Network Open and Goyal et al in Academic Emergency Medicine show low delay rates. Please modify the text to incorporate the generally low delay rates published in contemporary cohorts.

Response:

Thank you for pointing out this critical issue.
Additionally, we thanked for the educational review, including the literatures.

In response to your suggestion, we have added a description in discussion part as follows:

*[P15 L283-292]*

*On the other hand, a recent large cohort study in pediatrics reported a delay rate of <5% in the diagnosis of appendicitis [8, 41]. Michelson. et al. reported a 63% delay in appendicitis diagnosis in a study that included only patients in whom a revisit led to the appendicitis diagnosis [42]. This large difference in the frequency of diagnostic errors for appendicitis may be influenced by differences in the definition of diagnostic error, as well as differences in context, including clinical practice and differences in health care systems [43]. We believe that the frequency of delayed diagnosis in our study was higher than the rate reported in previous studies because we used strict the criteria for timely diagnosis to reduce bias, including only cases with a correct diagnosis made at the time of an initial visit as timely diagnosis.*
